# Supplementary figures and images for: Optical Fiber-Based Recording of Climbing Fiber Ca2+ Signals in Freely Behaving Mice
Source: Biology (Basel). 2022 Jun 13;11(6):907. doi: 10.3390/biology11060907 (PMC9220032; doi:10.3390/biology11060907)

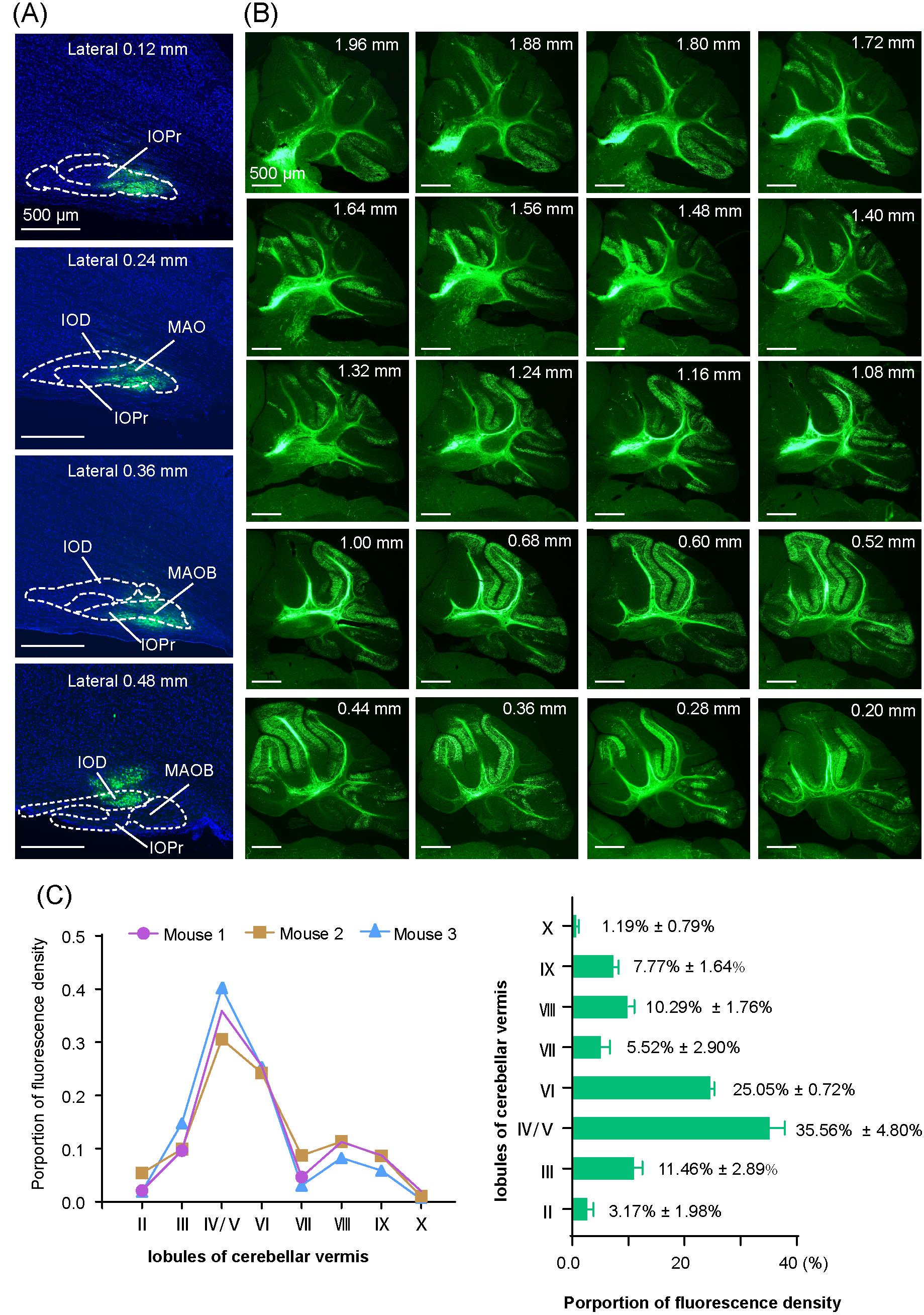

Supplement: Supplementary file 1 [file biology-11-00907-s001.zip › Figure S1.jpg]
